# Supplementary material for: Current-induced manipulation of exchange bias in IrMn/NiFe bilayer structures
Source: Nat Commun. 2021 Nov 5;12:6420. doi: 10.1038/s41467-021-26678-x (PMC8571404; doi:10.1038/s41467-021-26678-x)
Supplement: Supplementary file 1 — Supplementary Information [file 41467_2021_26678_MOESM1_ESM.docx]

**
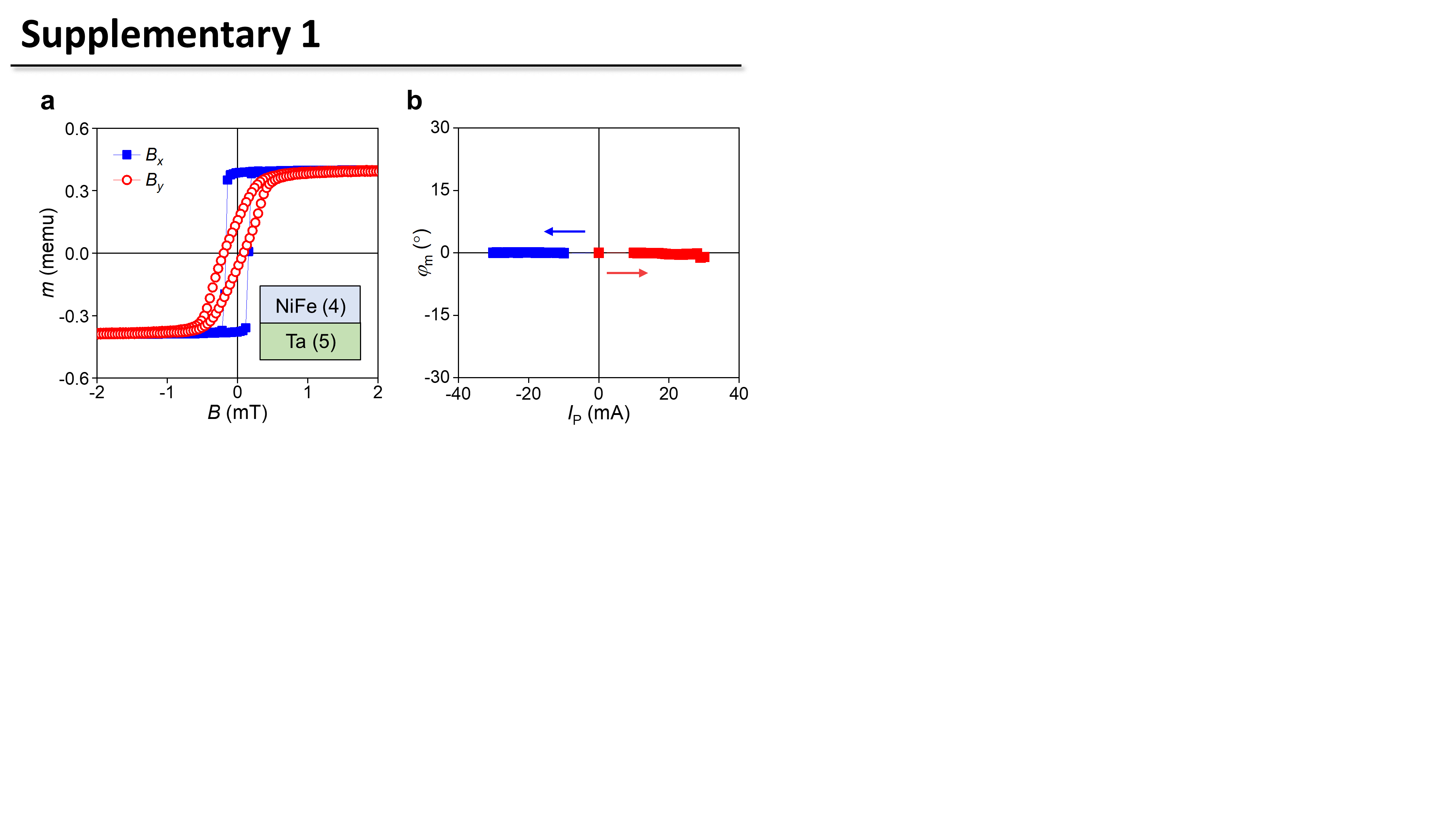
SUPPLEMENTARY INFORMATION**

**Current-induced manipulation of exchange bias in IrMn/NiFe bilayer structures**

Jaimin Kang^1^, Jeongchun Ryu^1,*^, Jong-Guk Choi^1^, Taekhyeon Lee^2^, Jaehyeon Park^2^_,_ Soogil Lee^1^, Hanhwi Jang^1^, Yeon Sik Jung^1^, Kab-Jin Kim^2^, and Byong-Guk Park^1,*^

^1^ *Department of Materials Science and Engineering and KI for Nanocentury, KAIST, Daejeon 34141, Korea*

*^2^ Department of Physics, KAIST, Daejeon 34141, Korea*

* Corresponding authors: Jeongchun Ryu ([jcryu@kaist.ac.kr](mailto:jcryu@kaist.ac.kr)) & Byong-Guk Park ([bgpark@kaist.ac.kr](mailto:bgpark@kaist.ac.kr))

**Supplementary Note 1. Material parameters.**


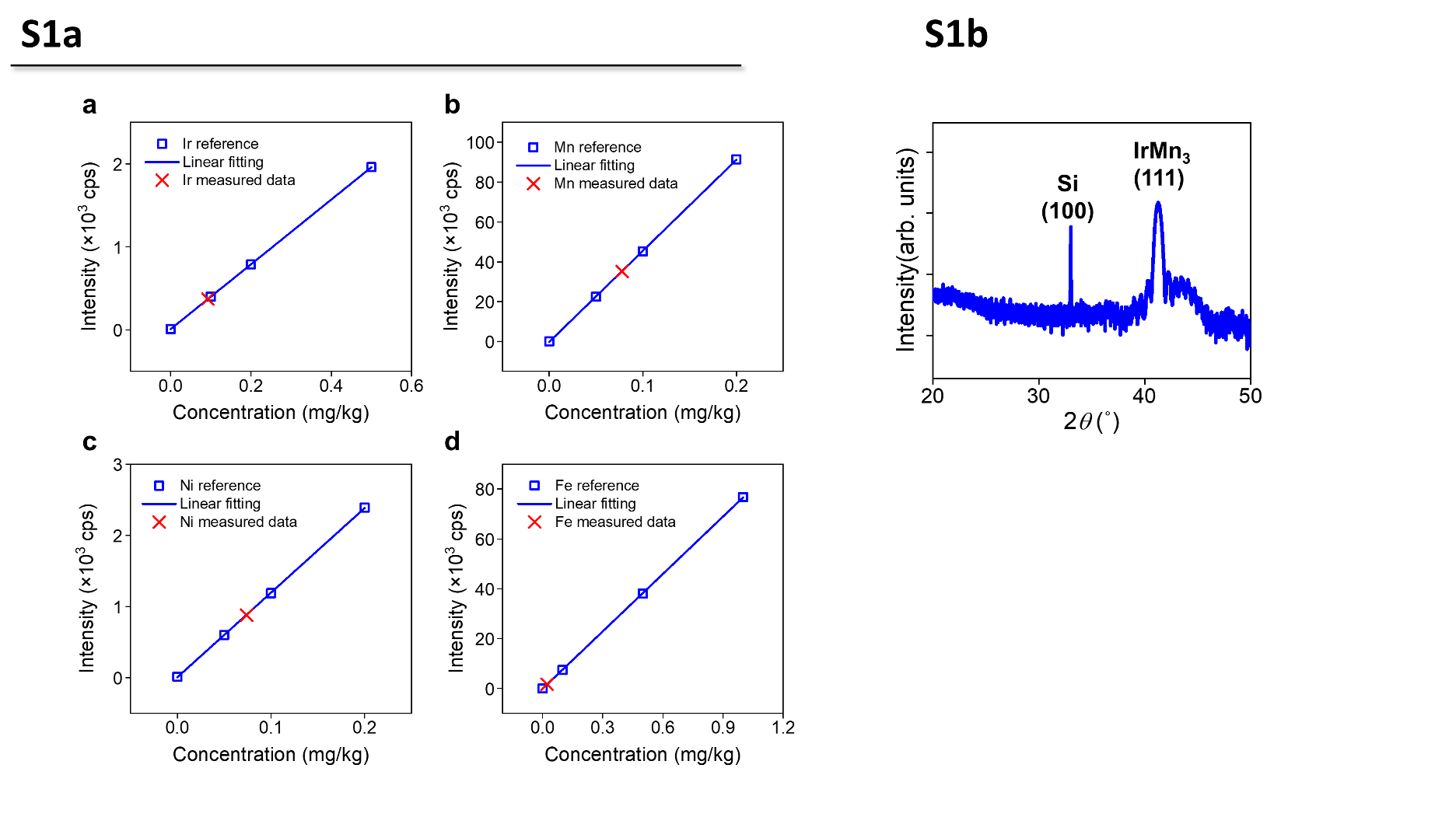
The composition was measured using an IrMn (5 nm)/NiFe (4 nm) sample with inductively-coupled plasma optical emission spectroscopy (ICP-OES). Figures S1a-d show the intensities of Ir, Mn, Ni, and Fe along with the calibration line, which correspond to 0.093 mg/kg, 0.078 mg/kg, 0.074 mg/kg and 0.022 mg/kg, respectively. By considering the atomic mass of each material, we obtained the atomic composition of the films, which is Ir_25_Mn_75_ and Ni_76_Fe_24._ Figure S2 shows X-ray diffraction (XRD) patterns for the IrMn (15 nm)/NiFe (4 nm)/MgO (3.2 nm)/Ta (2 nm) structure deposited on the Si substrate, demonstrating two peaks at 33˚ and 41.3˚, corresponding to the Si (200) plane and IrMn (111) plane, respectively.

**Figure S1 | Inductively-coupled plasma optical emission spectroscopy (ICP-OES) of the IrMn (5 nm)/NiFe (4 nm) sample. a-d**, ICP-OES intensities of Ir (**a**), Mn (**b**), Ni (**c**), and Fe (**d**). Blue squares and lines denote the reference intensity and a linear fitting line, respectively.

*
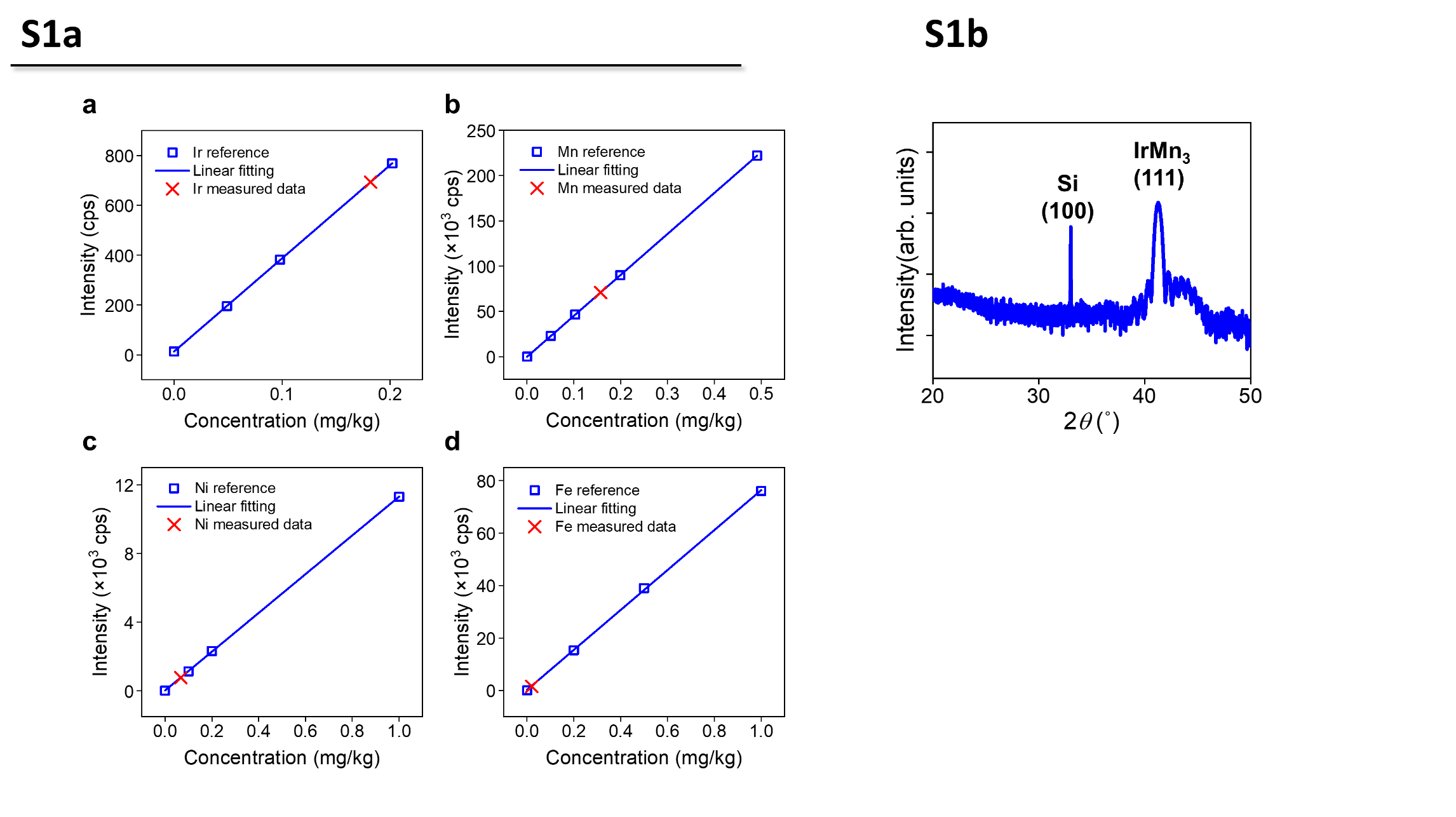
***Figure S2 | X-ray diffraction pattern of the IrMn (15 nm)/NiFe (4 nm)/MgO (3.2 nm)/Ta (2 nm) sample** **deposited on a Si substrate**.

**Supplementary Note 2. AMR measurements with different exchange bias directions**

We performed AMR measurements of the IrMn (5 nm)/NiFe (4 nm) sample, in which the exchange bias direction is initialized by an in-plane current pulse as *φ*_EB_ = 0˚, *φ*_EB_ = +15˚, and *φ*_EB_ = -15˚. The AMR curve along *B_x_* clearly shows that the center of the AMR curve of the sample with *φ*_EB_ = 0˚ shifts in the negative field direction (Fig. S3a), demonstrating an exchange bias along the positive *x*-direction. For the samples with *φ*_EB_ =±15, we also observe shifts in the negative field direction, but their magnitude is smaller than those with *φ*_EB_ = 0˚, which is due to the reduced *x*-component of the exchange bias for the samples with *φ*_EB_ =±15. Next, we measure the AMR while sweeping a magnetic field transverse to the current direction *B_y_* (Figure S3b). No shift is observed when *φ*_EB_ = 0˚, which is expected because the exchange bias is developed in the *x*-direction. On the other hand, we find that the AMR loop shifts in the opposite directions: negative (positive) when *φ*_EB_ = +15˚ (*φ*_EB_ = -15˚), demonstrating that the *y*-component (or rotation angle) of the exchange bias field has the opposite sign.

**
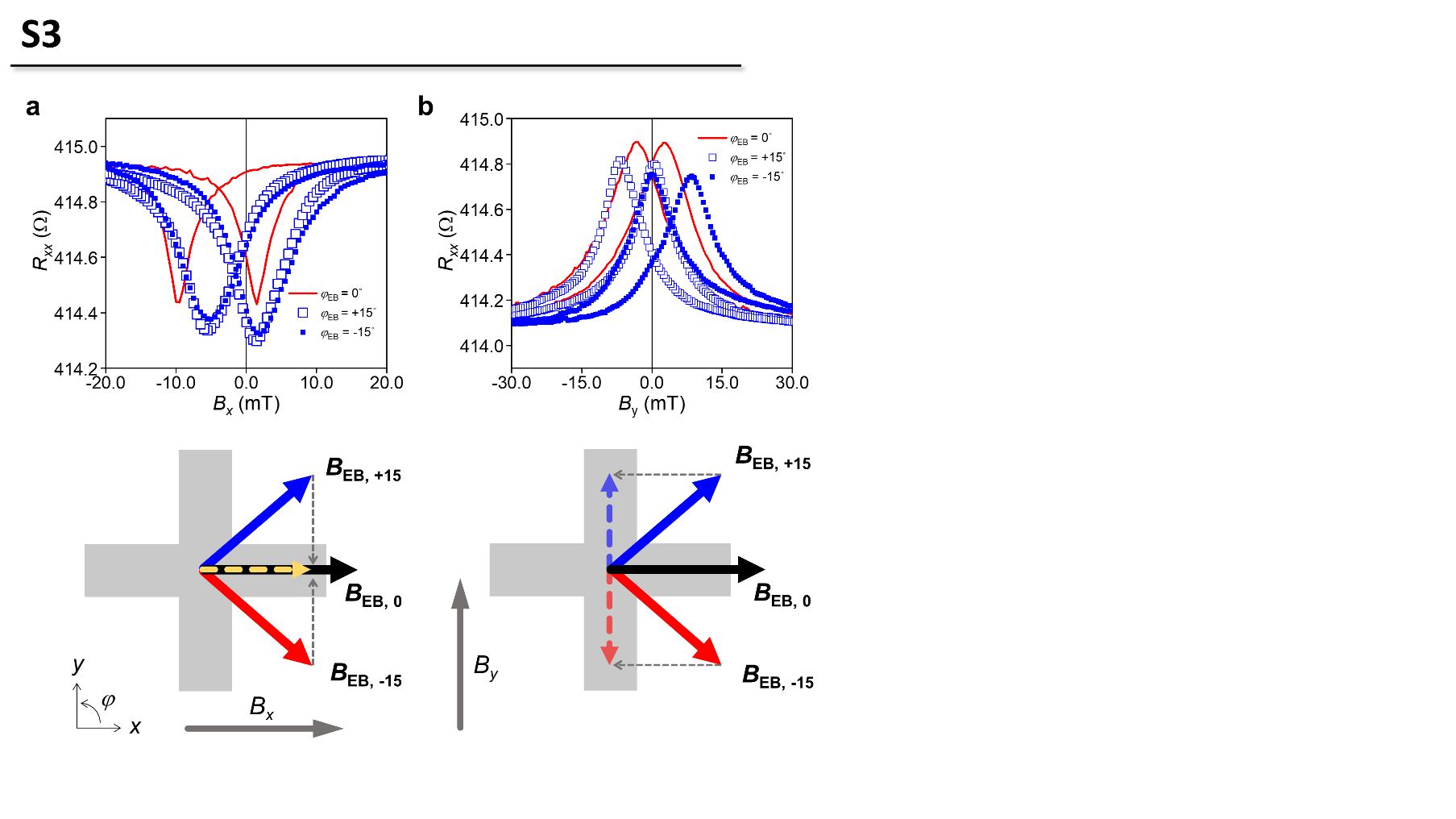
Figure S3 | AMR measurement with different exchange bias directions. a,b,** AMR measurements along the *x*-direction (**a**) and *y*-direction (**b**) of the IrMn (5 nm)/NiFe (4 nm) sample with a dc reading current of 100 μA. The line and symbols indicate the different samples with *φ*_EB_ = 0˚ (red lines), *φ*_EB_ = +15˚ (blue open squares), *φ*_EB_ = -15˚ (blue solid squares). Schematics describe the AMR measurements of the samples with different *φ*_EB_’s while sweeping magnetic fields along *B_x_* (left) and *B_y_* for (right). The solid arrows indicate the direction of the *B*_EB_ and the dotted arrows denote the *x*- (left) and *y*- (right) component of the *B*_EB_. Here the initial *φ*_EB_ of ±15˚ is set by a current pulse of $\mp$8.4×10^11^A/m^2^.

**Supplementary Note 3. Measurements of planar Hall effect (PHE) of an IrMn/NiFe bilayer and a NiFe single layer.**

**
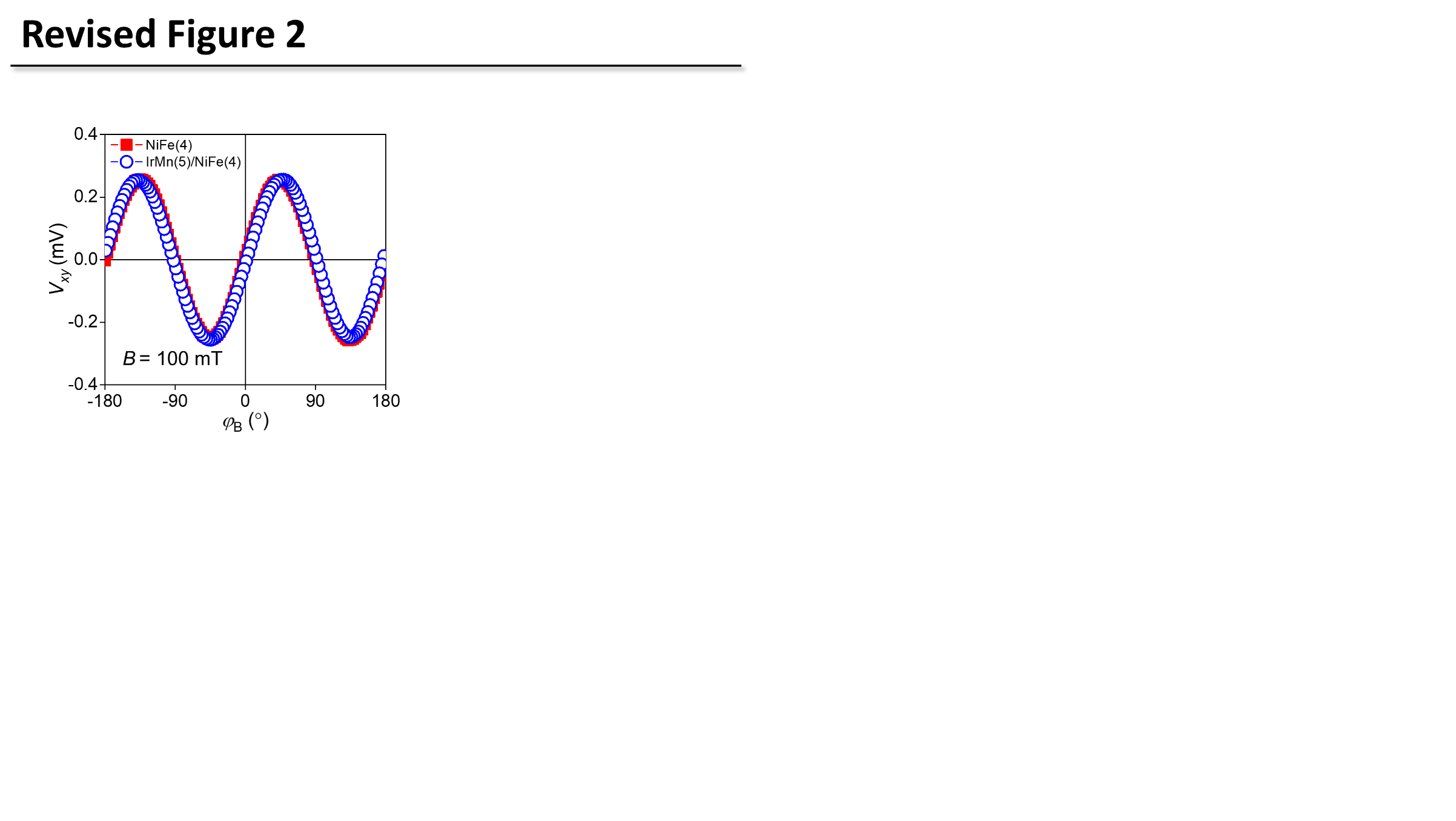
**To clarify if the *R*_H_ of the IrMn/NiFe sample is mainly dominated by the NiFe layer, we measure the planar Hall effect (PHE) of two samples: an IrMn (5 nm)/NiFe (4 nm) bilayer and a NiFe (4 nm) single layer (Fig. S4), where the same current density flowing in the NiFe layer is used. This shows that there is no significant difference in PHE values between the two samples, demonstrating negligible contribution of the IrMn layer to the *R*_H_ of the IrMn/NiFe sample.

**Figure S4 | Planar Hall effect (PHE) of an IrMn (5 nm)/NiFe (4 nm) bilayer and a NiFe (4 nm) single layer.** The reading current is 1 mA (0.83 mA) for the IrMn/NiFe bilayer (NiFe layer).

**Supplementary Note 4. SOT-induced exchange bias switching in various IrMn/FM structures**

We investigate other exchange bias systems such as IrMn/FM (CoFeB, CoFe, Ni) structures. Figure S5a shows the hysteresis loop of the IrMn (13 nm)/CoFeB (4 nm) sample, demonstrating the exchange bias. Note that the exchange bias of the IrMn/CoFeB (4nm) structure is not formed when IrMn thickness is smaller than 13 nm. Figure S5b shows the planar Hall resistance (*R*_H_) of the sample measured with an external magnetic field of 200 mT. We then performed current-induced SOT switching experiments using the same measurement procedure of the main text (Fig. S5c). The result shows a similar switching behaviour to that of the IrMn/NiFe structure, but with a smaller rotation angle (*ϕ*_EB_ = ±3˚). The small effect may be due to the thick IrMn layer requiring a larger current to generate the SOT. We also find similar results in IrMn (12 nm)/CoFe (4nm) [Fig. S5d-f] and IrMn (5 nm)/Ni (4 nm) structures [Figs. S5g-i], which exhibits *ϕ*_EB_ = ±9˚ and *ϕ*_EB_ = ±8˚, respectively.

**
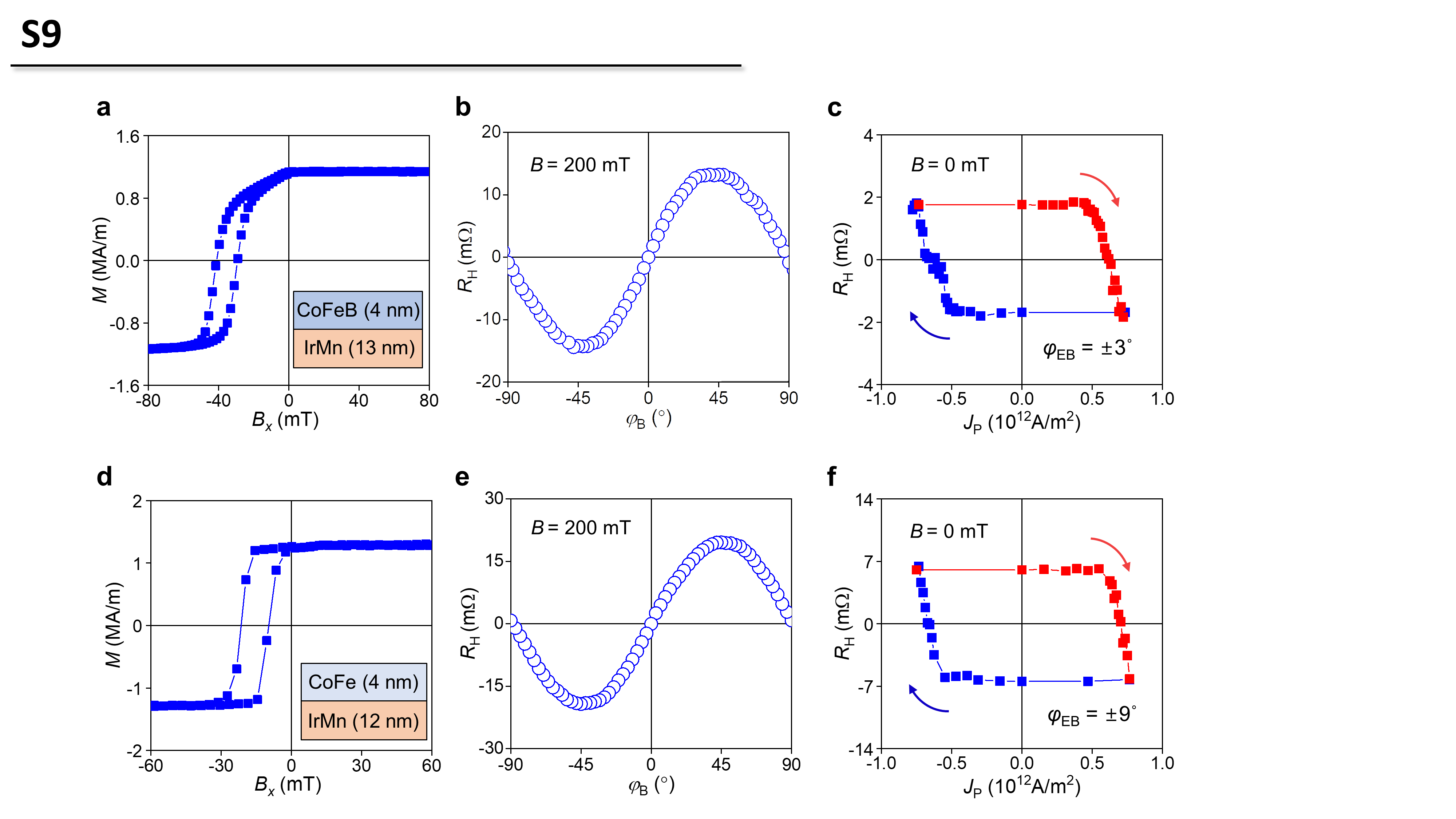

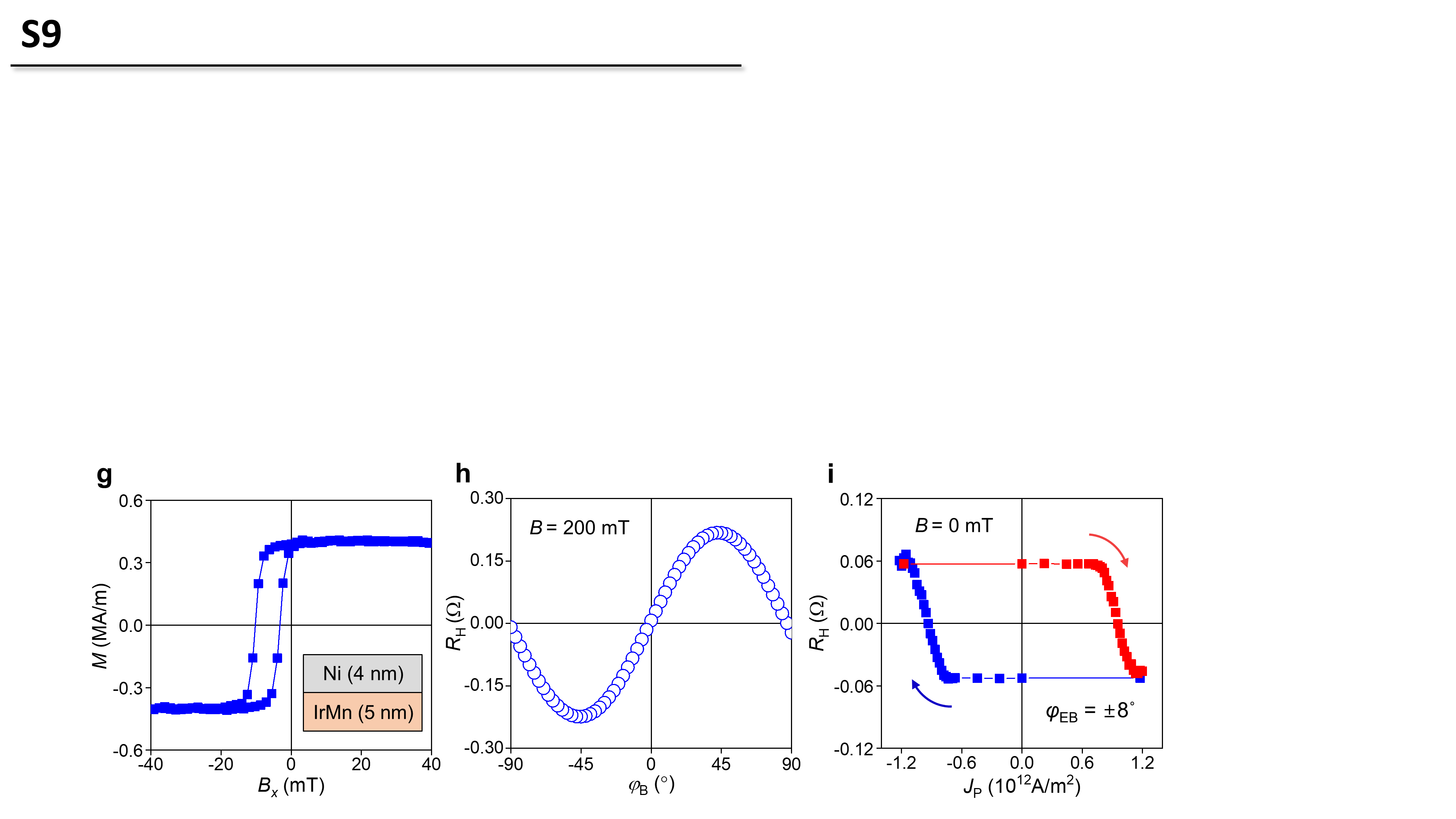
Figure S5 | SOT-induced exchange bias switching in various IrMn/FM structures**. a, Hysteresis loop measured with a magnetic field along the *x*-axis (*B_x_*) in the IrMn (13 nm)/CoFeB (4 nm) sample. **b,** *R*_H_ versus azimuthal angle of a magnetic field (*ϕ*_B_) of 200 mT of the IrMn/CoFeB sample. **c,** The *R*_H_ vs *J*_P_ curves of the IrMn/CoFeB sample, where the arrows denote the sweeping direction of *J*_P_. **d-f,** Hysteresis loop (**d**), *R*_H_ versus *ϕ*_B_ curve (**e**), *R*_H_ vs *J*_P_ curves (**f**) of the IrMn (12 nm)/CoFe (4 nm) sample. **g-i**, Hysteresis loop (**g**), *R*_H_ versus *ϕ*_B_ curve (**h**), *R*_H_ vs *J*_P_ curves (**i**) of the IrMn (5 nm)/Ni (4 nm) sample.


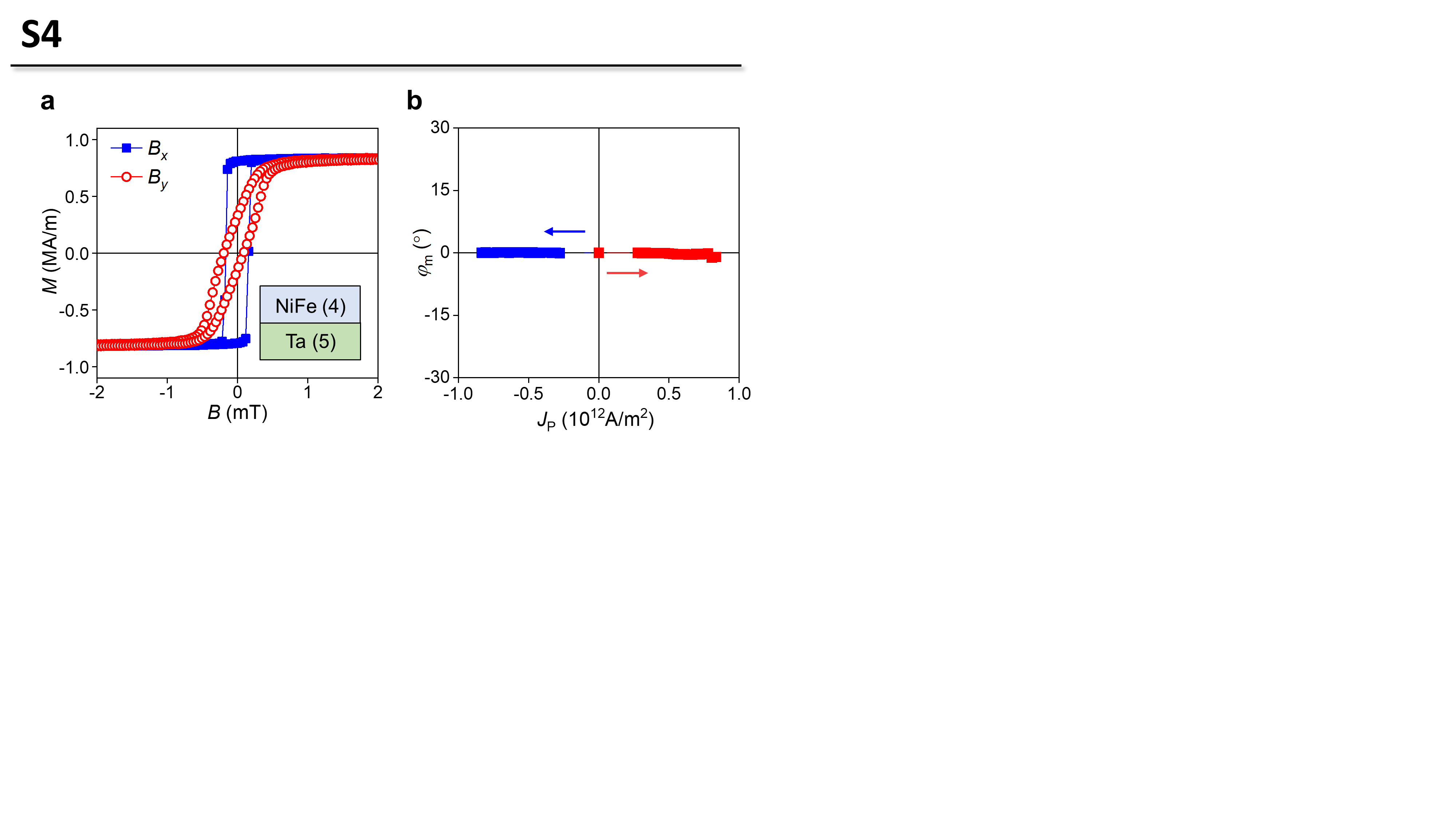
**Supplementary Note 5. Hysteresis loop and switching measurement in a Ta/NiFe structure**

Figure S6 | Hysteresis loop and switching measurement in a Ta/NiFe structure. a, Hysteresis loops of the Ta (5nm)/NiFe (4 nm) structure measured with magnetic fields along the *x*-axis (solid blue) and *y*-axis (open red). b, *R*_H_ versus in-plane current pulse (*J*_P_) of the Ta/NiFe bilayer. The arrows denote the sweeping direction of *J*_P_.

**Supplementary Note 6. Thermal conductivity of the Si substrate**


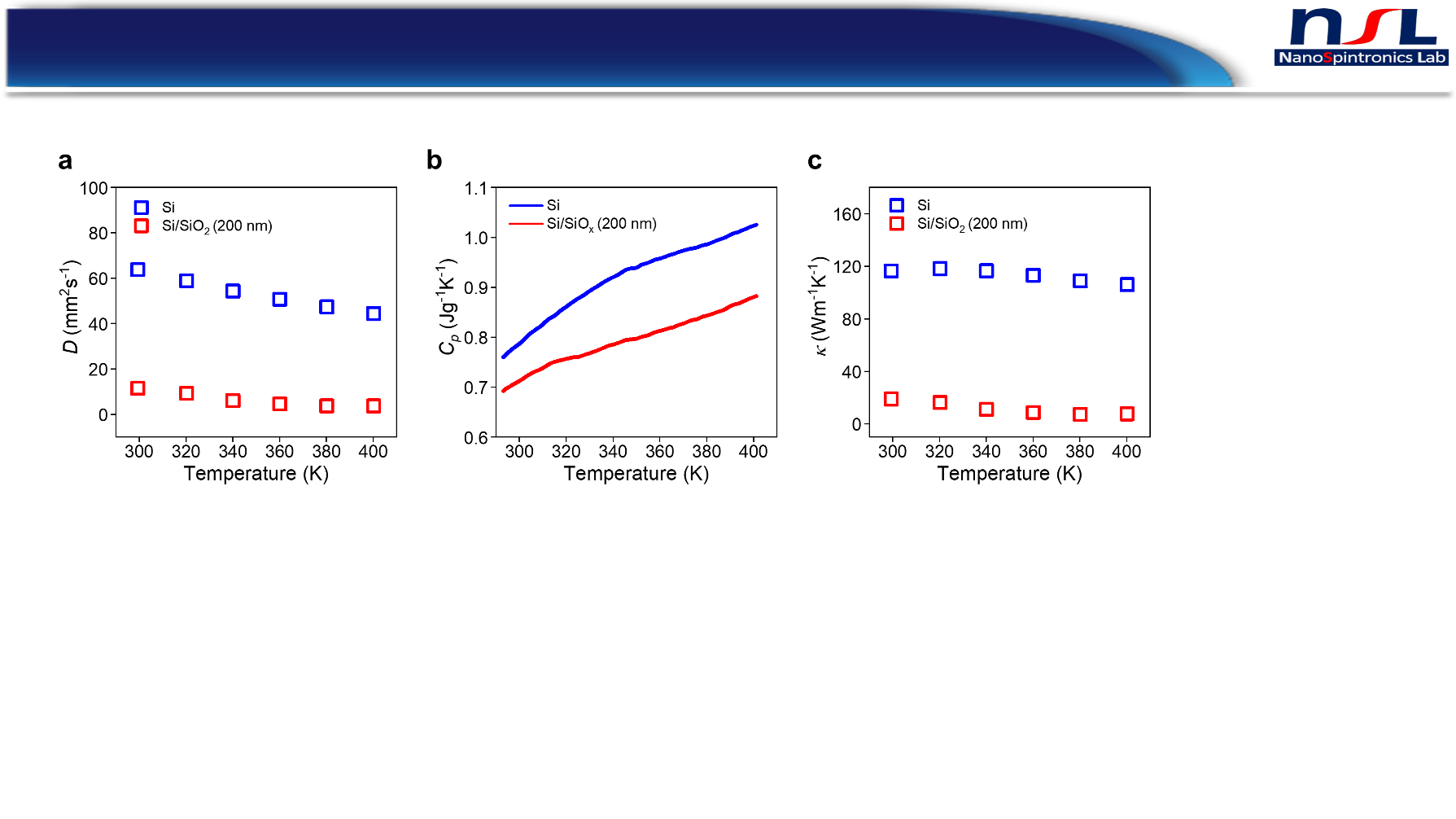
We measured the thermal conductivity of the Si substrate, which is compared to that of the Si/SiO_2_ (200 nm) substrate. The thermal conductivity of a material is given by *κ*=*ρC*_p_*D*, where *ρ*, *C*­_p_, and *D* is the bulk density, the specific heat capacity, and the thermal diffusivity, respectively. We first measured the temperature-dependent thermal diffusivity (*D*) of the substrates using the laser flash method [S1]. Figure S7a shows a gradual decrease in *D* as the temperature increases from 300 K to 400 K, which is attributed to increased phonon–phonon scattering at high temperatures [S2]. Then, we measured the specific heat capacity (*C_p_*) of the substrates by differential scanning calorimetry as shown in Fig. S7b. We finally obtained the thermal conductivity (*к*) of the Si and Si/SiO_2_ substrates (Fig. S7c) by considering the measured *D*, *C_p_* (Fig. S7a,b), and *ρ* of 2.3g/cm^3^ determined by Archimedes method. This shows that the *к* of the Si substrate is ~116.5 Wm^-1^K^-1^, which is in good agreement with the literature value of 124 Wm^-1^K^-1^ [S3]. On the other hand, the Si/SiO_2_ substrate has much smaller *к* values than the Si substrate for all measured temperatures.

**Figure S7 | Temperature-dependent thermal conductivities of the substrate. a-c,** Temperature-dependent thermal properties of the Si and Si/SiO_2_ (200 nm) substrates: thermal diffusivities (**a**), specific heat capacities (**b**), and thermal conductivities (**c**).

**Supplementary Note 7. Blocking temperature of the IrMn/NiFe structure**

To determine the blocking temperature of the IrMn (5 nm)/NiFe (4 nm) sample, we measured magnetization curves of the IrMn/NiFe sample at various temperatures ranging from 100 K to 400 K (Figures S8a,b). The exchange bias field (*B*_EB_) that is determined by the hysteresis shift disappears at a temperature greater than 385 K, which is the blocking temperature of the IrMn (5 nm)/NiFe (4 nm) sample.


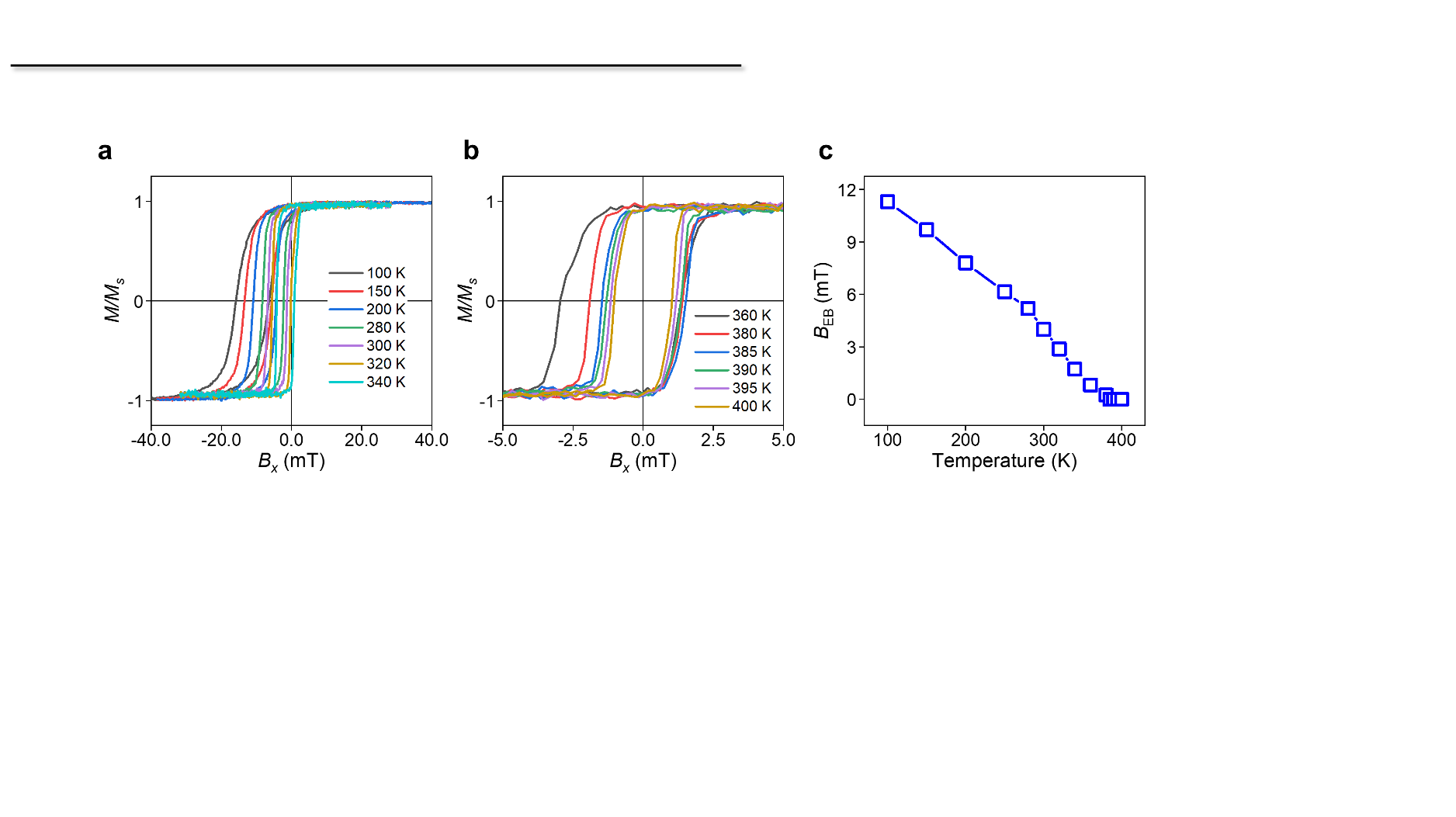


**Figure S8 | Temperature dependent magnetization curves of the IrMn (5 nm)/NiFe (4 nm) sample. a-b,** Magnetization loops measured at various temperatures of 100~340 K (**a**) and 360~400 K (**b**).

**Supplementary Note 8. Current-induced switching experiment at a low temperature**


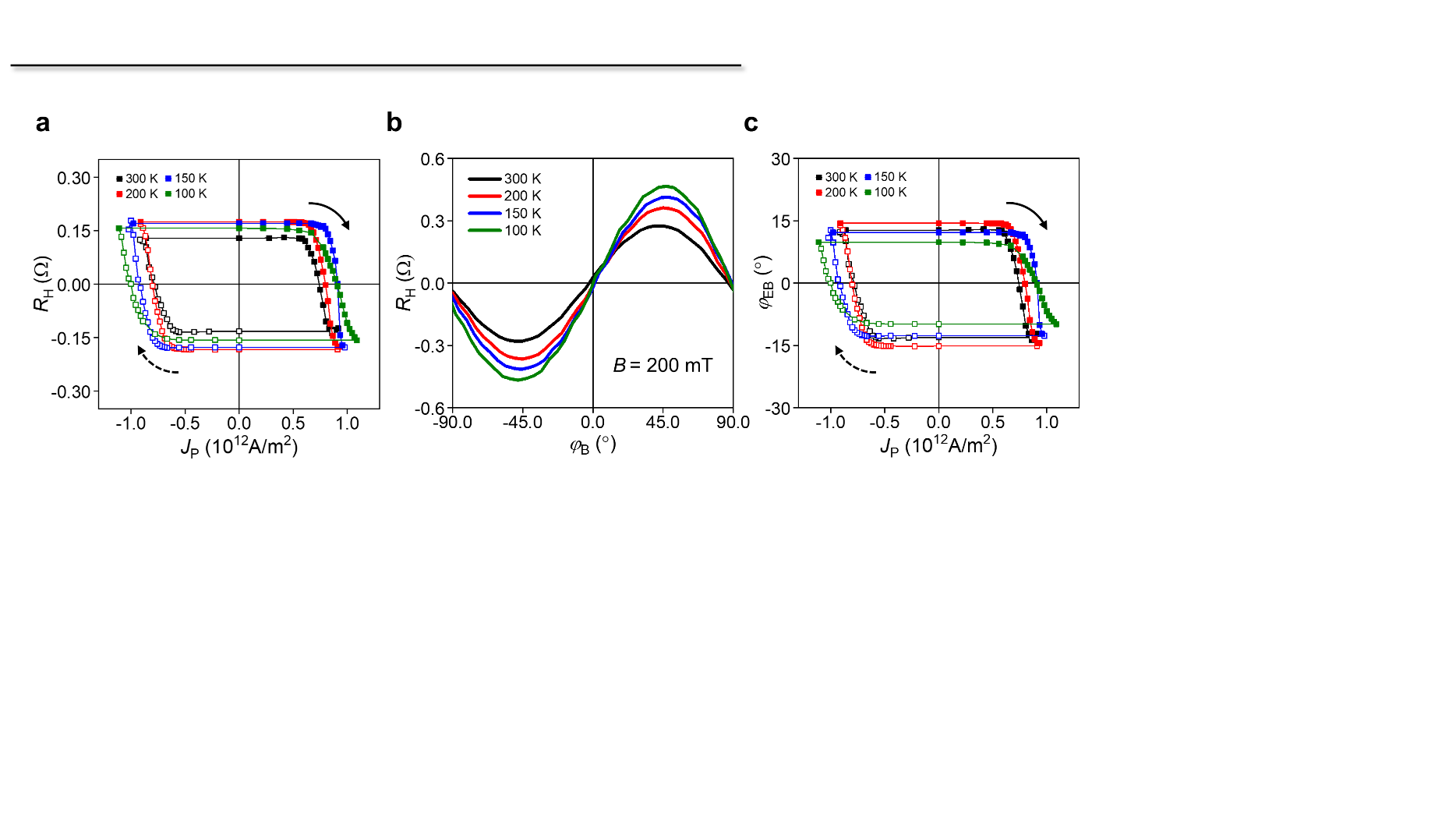
 To further elucidate the Joule heating effect, we examine the current-induced manipulation of the exchange bias direction of the IrMn (5 nm)/NiFe (4 nm) sample at low temperatures. We performed current-induced exchange bias switching experiment at temperatures between 100 K and 300 K using the same measurement protocols described in the main text. Figure S9a shows the switching results of *R*_H_ versus pulse current *J*_P_, demonstrating the switching behaviour for all measuring temperatures is similar to that measured at 300 K (Fig. 4b of the main text). By comparing the maximum *R*_H_ value of each temperature with planar Hall resistance measured under an external magnetic field of 200 mT (Fig. S9b), we extract the rotation angle of the exchange bias (*φ*_EB_) for each temperature (Fig. S9c). This demonstrates that the current-induced modulation of *φ*_EB_ is obtained regardless of the measuring temperature down to 100 K, which is much lower than the blocking temperature. This result confirms that the Joule heating effect is not a possible origin of the current-induced manipulation of exchange bias.

Figure S9 | Current-induced manipulation of the exchange bias at low temperatures. a, The Hall resistance (*R*_H_) of the IrMn (5 nm)/NiFe (4 nm) sample measured after applying an in-plane current pulse *J*_P_ at various temperatures. b, Planar Hall resistance *R*_H_ versus azimuthal angle of a magnetic field (*ϕ*_B_) of 200 mT at different temperatures. c, The rotation angle of the exchange bias *φ*_EB_ versus *J*_P_ curves at various temperatures. The arrows denote the sweeping direction of *J*_P_.

**Supplementary Note 9. Oersted field by the injecting current**

We investigate if the Oersted field due to injecting current affects the current-induced modulation of the exchange bias. In this regard, we first estimate the current distribution of each layer by measuring the resistance *R_xx_* of the devices with different IrMn and NiFe thicknesses. Here, NiFe thickness *t*_NiFe_ ranges from 3 to 15 nm in IrMn (5 nm)/NiFe (*t*_NiFe_) structures and IrMn thickness *t*_IrMn_ ranges from 5 to 25 nm in IrMn (*t*_IrMn_)/NiFe structures. Figure S10a shows the (1*/R_xx_*)·*L*/*W* values of the samples as a function of *t*_NiFe_ and *t*_IrMn_, where *L* and *W* is the length and width of the Hall-bar device, respectively. From the results, we extract the resistivity of each layer; 316 μΩcm for IrMn and 51.3 μΩcm for NiFe layer. This indicates that in the NiFe (4 nm)/IrMn (5 nm) bilayers, 83.1% and 16.9% of the current flows through the NiFe and IrMn layers, respectively. Moreover, in the IrMn (5 nm)/NiFe (4 nm)/Ta (1.5 nm) sample, where the resistivity of Ta is 353.8 μΩcm, 16.2%, 79.5%, 4.3% of the current flows through the IrMn, NiFe, and Ta (1.5 nm) layers, respectively.

Taking the current distributions into account, we calculated an Oersted field acting on NiFe layer in the IrMn (5 nm)/NiFe (4 nm) and IrMn (5 nm)/NiFe (4 nm)/Ta (1.5 nm) samples using equations S8-S12 in Supplementary Information 6 of the reference [S4]. Figure S10b,c shows the Oersted fields along the *y* and *z* direction (*B*^Oe,^*^y^* and *B*^Oe,^*^z^*) generated in the middle of the NiFe layer of the samples. Here, we use a current density of 7.4×10^11^A/m^2^, which is the switching current density of the sample. It is found that *B*^Oe,^*^y^* is ~0.68 mT in the IrMn (5 nm)/NiFe (4 nm) sample and ~0.55 mT in the IrMn/NiFe/Ta sample.

*
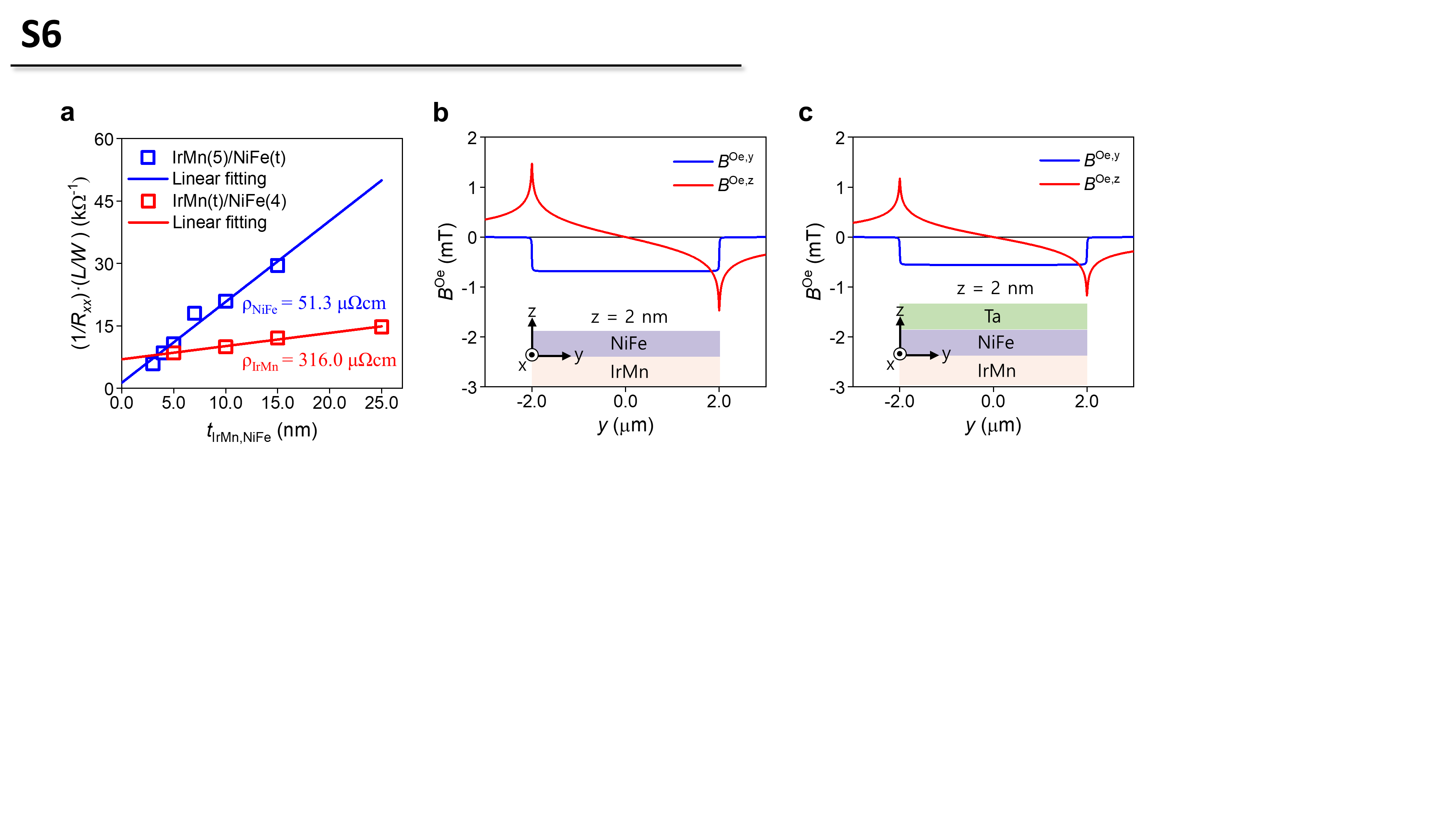
***Figure S10 | Current distribution and Oersted field. a,** (1*/R_xx_*)·(*L*/*W*) as a function of NiFe thickness *t*_NiFe_ or IrMn thickness *t*_IrMn_ in IrMn/NiFe bilayer structures. **b,c** Calculated Oersted fields along the *y* and *z* direction (*B*^Oe,^*^y^* and *B*^Oe,^*^z^*) in the middle of NiFe layer of the IrMn (5 nm)/NiFe (4 nm) sample (**b**) and the IrMn (5 nm)/NiFe (4 nm)/Ta (1.5 nm) sample (**c**). Here, a current density is 7.4×10^11^A/m^2^.

**Supplementary Note 10. Spin Hall angle of Ta and IrMn**

To quantitatively estimate the spin Hall angles of Ta and IrMn, we performed in-plane harmonic measurements of the Ta (5 nm)/NiFe (4 nm) and IrMn (5 nm)/NiFe (4 nm) samples [S2]. Figures S11a,b show the 2^nd^ harmonic Hall resistance ($R^{2\omega}$) versus azimuthal angle (*φ*) curves of Ta/NiFe and IrMn/NiFe samples, which are measured with an ac current by rotating the samples under different external magnetic fields (*B*_ext_). Figures S11c,d show the cos*φ* component of $R^{2\omega}$ divided by $R_{\mathrm{AHE}}$ [$R_{\cos\varphi}^{2\omega}/R_{\mathrm{AHE}}$] as a function of 1/*B*_eff_. Since the *B*_DLT_ and the associated effective spin Hall angle ($\theta_{\mathrm{SH}}^{\mathrm{eff}}$) is represented by the slope of the $R_{\cos\varphi}^{2\omega}$/$R_{\mathrm{AHE}}$ versus 1/*B*_eff_ curves, the negative (positive) slope in the Ta/NiFe (IrMn/NiFe) sample indicates a negative (positive) $\theta_{\mathrm{SH}}^{\mathrm{eff}}$ of Ta (IrMn). The *B*_DLT_ is obtained to be -1.2±0.4 mT (2.3±0.5 mT) for the Ta/NiFe (IrMn/NiFe) sample at a current density of 1.0×10^11^A/m^2^. These values are comparable to the previously reported ones [S5,S6]. We also plot the (${2cos}^{3}\varphi-cos\varphi$) component of $R^{2\omega}$ divided by ${2R}_{\mathrm{PHE}}$ $[R_{{2cos}^{3}\varphi-cos\varphi}^{2\omega}/{2R}_{\mathrm{PHE}}]$as a function of 1/*B*_ext_, of which slope represents the combination of the *B*_FLT­_ and *B*_Oe_. The extracted *B*_FLT­_ + *B*_Oe_ is 0.05±0.06 mT (0.13±0.01 mT) for the Ta/NiFe (IrMn/NiFe) sample at a current density of 1.0×10^11^A/m^2^. This demonstrates that the *B*_FLT­_ of the two samples is negligibly small as compared to *B*_DLT_.


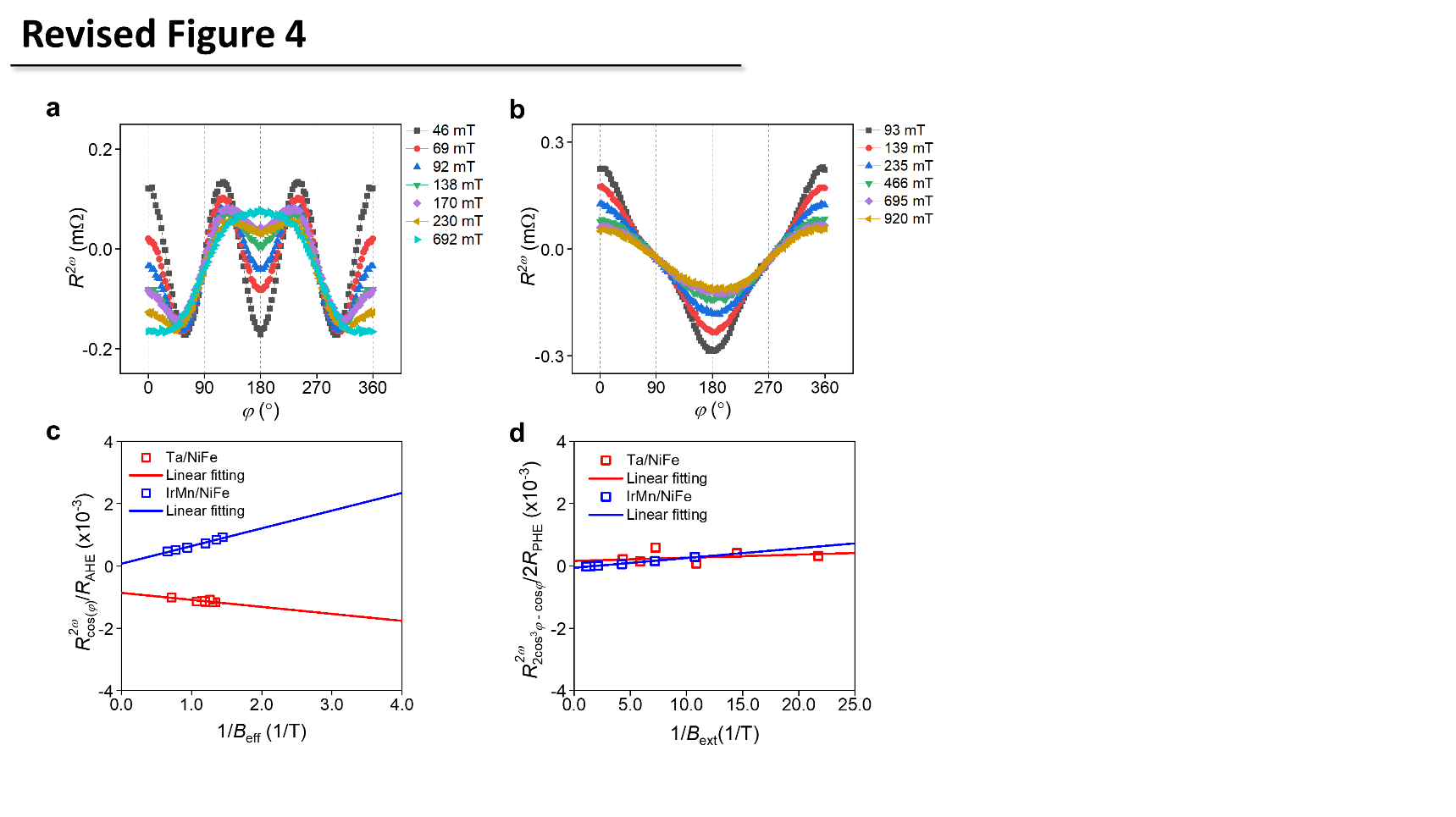
**Figure S11 | In-plane harmonic spin-orbit torque measurements in Ta/NiFe and IrMn/NiFe structures.** **a,b** Azimuthal angle (*φ*) dependent 2^nd^ harmonic Hall resistance, *R*^2w^(*φ*), measured under a different *B*_ext_ in the Ta (5 nm)/NiFe (4 nm) (**a**) and IrMn (5 nm)/NiFe (4 nm) (**b**) samples. **c,d,** The extracted $\varphi$-dependent components of $\text{R}_{\text{xy}}^{\text{2ω}}$; $\cos\varphi$ component versus 1/*B*_eff_ (**c**), ($2\cos^{3}\varphi-cos\varphi)$ component versus 1/*B*_ext_ (**d**).

**Supplementary Note 11. NiFe thickness dependence of SOT-induced exchange bias switching**

**
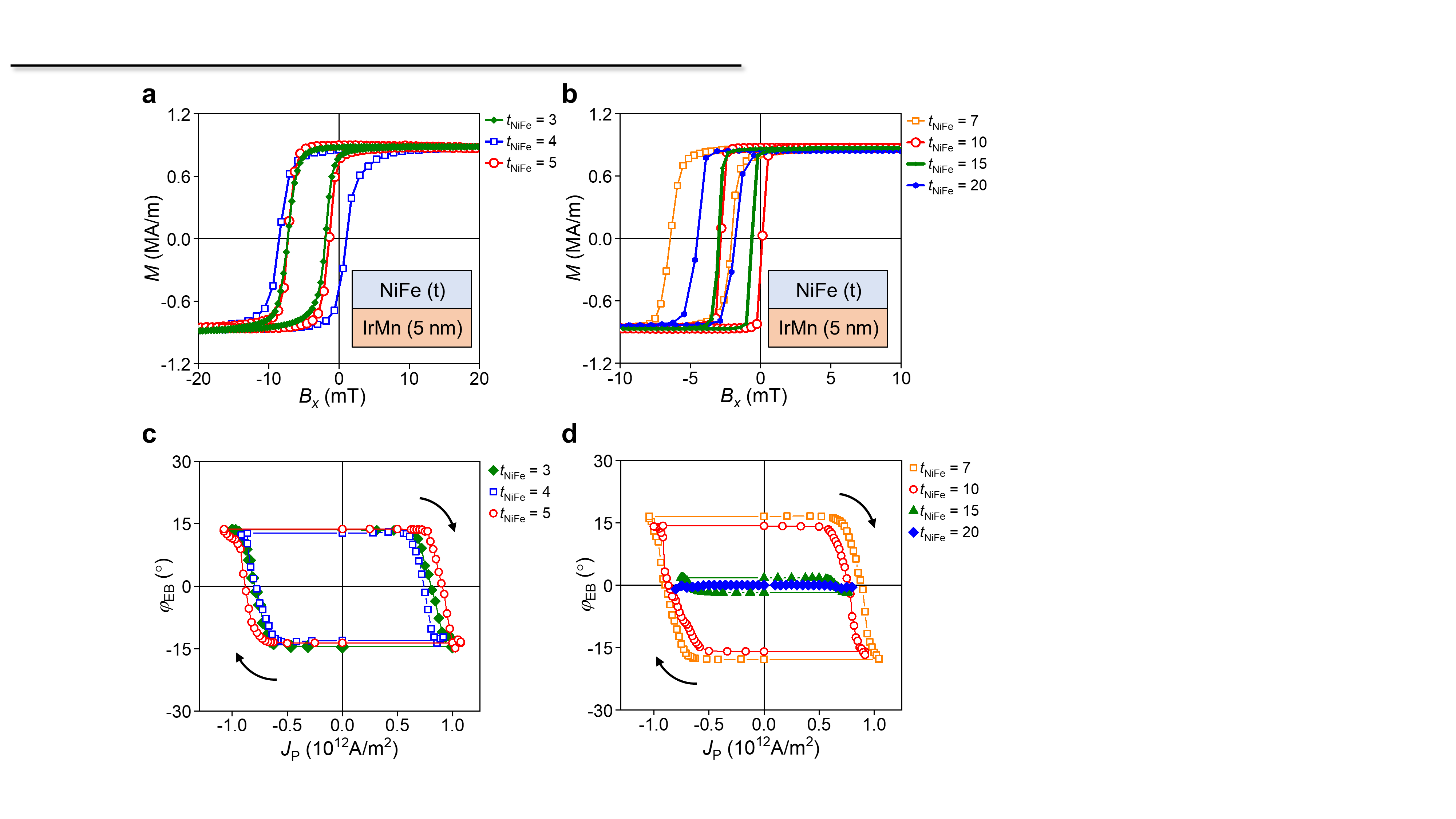
**

**Figure S12 |** **NiFe thickness dependence of SOT-induced exchange bias switching. a-b**. Hysteresis loop measured using a magnetic field along the *x*-axis, *B_x_* for *t*_NiFe_ = 3 nm to 5 nm (**a**), for *t*_NiFe_ = 7 nm to 20 nm (**b**). The *ϕ*_EB_ versus current density (*J*_P_) curves, where the arrows denote the sweeping direction of *J*_P_.

**Supplementary Note 12. Memristive behaviour of exchange bias switching with the opposite current sweep**

**
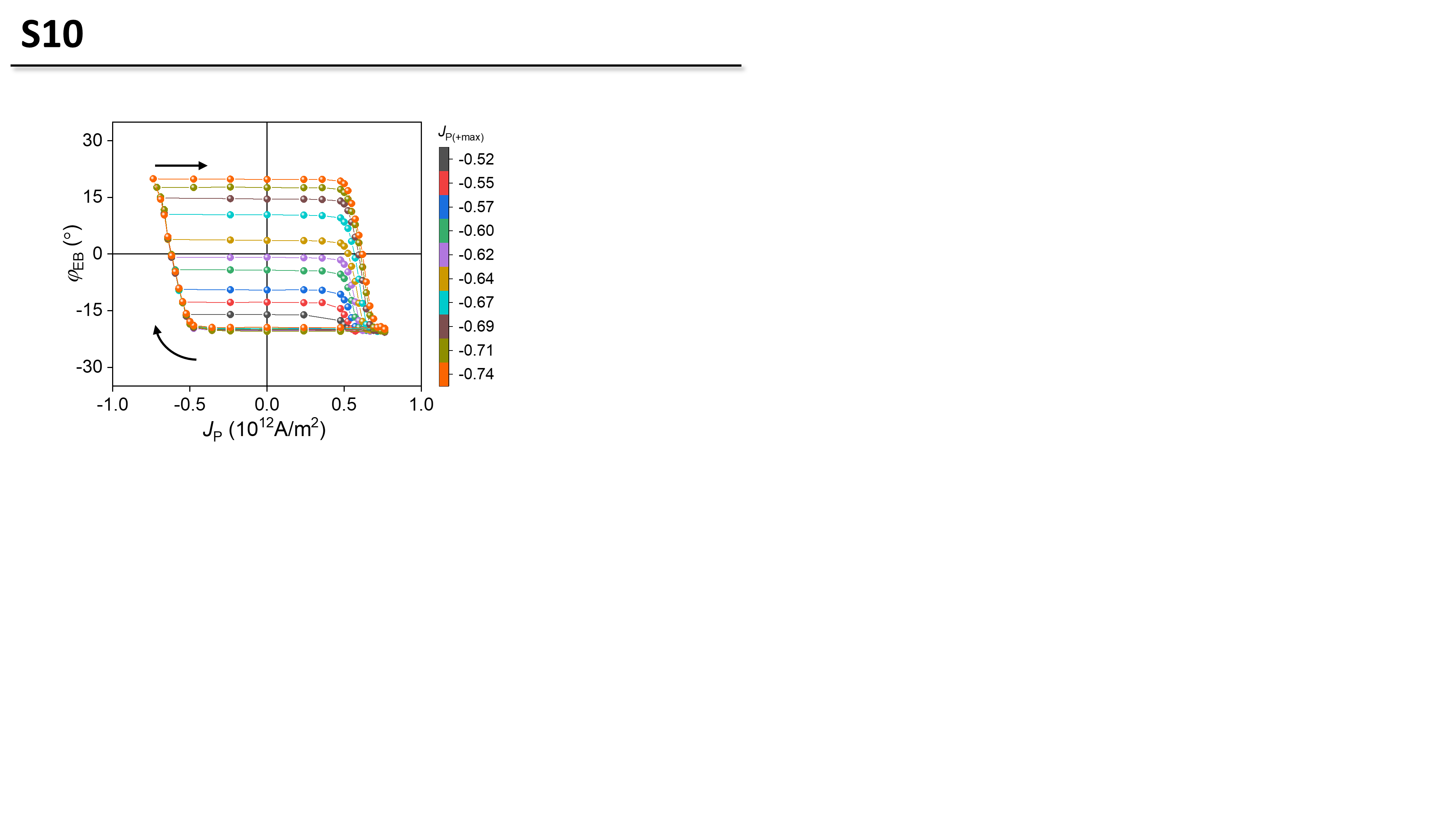
**

**Figure S13 | Memristive behaviour of exchange bias switching with the opposite current sweep.** Minor *φ*_EB_ vs *J*_P_ curves for the IrMn (5 nm)/NiFe (4 nm)/Ta (1.5 nm) sample with a 4-μm-wide Hall bar, measured under the same sequence as shown in Fig. 5 of the main text with *J*_P,ini_ = +7.4×10^11^ A/m^2^ and *J*_P(-max)_ varying from -5.2×10^11^ A/m^2^ to -7.4×10^11^ A/m^2^.

**Supplementary References**

1. Min, S. et al. A new laser flash system for measurement of the thermophysical properties. *Thermochim. Acta* **455**, 46 (2007)
2. Gurunathan, R. et al. Analytical models of phonon–point-defect scattering. *Phys. Rev. Appl.* **13**, 034011 (2020)
3. **Cardarelli**, F. *Materials handbook*. (Springer-Verlag London, 2008)
4. Baumgartner, M. et al. Spatially and time-resolved magnetization dynamics driven by spin-orbit torques. *Nat. Nanotechnol.* **12**, 980-986 (2017)
5. Avci, C. et al. Interplay of spin-orbit torque and thermoelectric effects in ferromagnet/normal-metal bilayers. *Phys. Rev. B* **90**, 224427 (2014)
6. Oh, Y.-W. et al*.* Field-free switching of perpendicular magnetization through spin-orbit torque in antiferromagnet/ferromagnet/oxide structures. *Nat. Nanotechnol.* **11**, 878–884 (2016).
